# Ultrasound Characteristics of an Isolated Redundant Foramen Ovale Flap in Fetuses and Its Differential Diagnosis from Aortic Coarctation

**DOI:** 10.3390/jcm15083166

**Published:** 2026-04-21

**Authors:** Liya Li, Yuxin Li, Shijie Zhang, Shaozheng He, Qiuyue Chen, Guorong Lyu

**Affiliations:** 1 Department of Ultrasound Medicine, Second Affiliated Hospital of Fujian Medical University, No. 34 North Zhongshan Road, Quanzhou 362000, China; lly7473@fjmu.edu.cn (L.L.); lyx951@fjmu.edu.cn (Y.L.); 1230920139@fjmu.edu.cn (S.Z.); hsz@fjmu.edu.cn (S.H.); qiuyuechen@fjmu.edu.cn (Q.C.); 2 Collaborative Innovation Center for Maternal and Infant Health Service, Application Technology of Education Ministry, Quanzhou Medical College, Quanzhou 362000, China

**Keywords:** fetal echocardiography, prenatal diagnosis, aortic coarctation, ultrasonography

## Abstract

**Objective:** To evaluate the potential utility of the ratio of the maximal diameter of the bulging foramen ovale flap to the left atrial diameter (FOFD/LAD) for distinguishing false-positive prenatal suspicion of fetal coarctation of the aorta (CoA), and to examine its association with fetal cardiac structural parameters. **Materials and Methods:** This retrospective study included a selected referral cohort of 44 fetuses with prenatal suspicion of CoA, who were classified postnatally into the false-positive prenatal suspicion group (*n* = 29) or the true CoA group (*n* = 15), along with 50 gestational age-matched controls. FOFD, LAD, atrial and ventricular diameters, great-vessel diameters, and aortic isthmus Z-scores were measured. Associations were assessed using Spearman rank correlation, and intergroup differences were evaluated using the Kruskal–Wallis test with Bonferroni-adjusted Mann–Whitney U tests. **Results:** FOFD/LAD was significantly higher in the false-positive prenatal suspicion group than in the true CoA and control groups (all *p* < 0.001), whereas no difference was observed between the true CoA and control groups (*p* = 0.059). In the false-positive and control groups, FOFD/LAD was positively associated with RAD/LAD, RVD/LVD, and PAD/AoD (all *p* < 0.001). Both suspected CoA groups showed higher right-to-left cardiac structural ratios and aortic isthmus Z-scores than controls (all *p* < 0.001), but these indices did not differ between false-positive prenatal suspicion and true CoA cases. **Conclusions:** In a selected cohort of fetuses with prenatal suspicion of CoA, an increased FOFD/LAD may reflect the presence of a redundant foramen ovale flap and may serve as a promising adjunctive parameter for distinguishing RFOF-related CoA mimicry from true CoA. However, given the limited sample size and moderate reproducibility, these findings should be considered exploratory and require validation in larger independent cohorts.

## 1. Introduction

The foramen ovale is a key component of fetal circulation, enabling oxygen-rich blood from the ductus venosus to bypass the right heart and preferentially perfuse the brain and myocardium [1,2]. When interatrial flow becomes restrictive, adverse neonatal outcomes may occur, including pulmonary hypertension and associations with extracardiac anomalies [3,4,5,6]. A redundant foramen ovale flap (RFOF) is an anatomic variant characterized by an elongated and highly mobile septum primum flap; when aneurysmal or prolapsing toward the mitral valve, it may intermittently impede interatrial streaming and reduce effective left-heart filling, potentially contributing to asymmetric cardiac development [7,8,9]. RFOF should be distinguished from a restrictive foramen ovale. Although both conditions may be associated with elevated left atrial pressure, a restrictive foramen ovale is typically characterized by a narrowed opening with high-velocity flow, whereas RFOF presents with a relatively wide orifice and a highly mobile flap [7].

Recent studies have suggested that RFOF can mimic prenatal echocardiographic findings that are commonly used to suspect coarctation of the aorta (CoA), including apparent left-heart hypoplasia and aortic arch narrowing [7,9]. Because most prenatal CoA prediction models rely heavily on right-to-left chamber size ratios and outflow-tract dimensions, RFOF may increase the rate of false-positive prenatal suspicion [10,11]. However, systematic comparisons of RFOF-related CoA mimicry and true CoA with respect to prenatal imaging and postnatal outcomes remain limited.

We aimed to characterize the prenatal ultrasound features of RFOF, assess its association with fetal cardiac structural indices, and explore whether the FOFD/LAD ratio may help differentiate RFOF-related CoA mimicry from true CoA among fetuses with prenatal suspicion of CoA.

## 2. Materials and Methods

### 2.1. Demographic and Clinical Data

This retrospective study included fetuses with prenatal suspicion of CoA identified on ultrasound screening at our institution between February 2015 and April 2025. Inclusion criteria were: (1) singleton pregnancy; (2) prenatal suspicion of CoA based on an overall fetal echocardiographic impression of right-sided predominance, including disproportion between the right and left cardiac chambers and/or great vessels, together with apparent narrowing of the aortic isthmus on the aortic arch view, particularly in comparison with the adjacent transverse arch at the level of the left subclavian artery; and (3) absence of maternal complications (preeclampsia, diabetes mellitus, hypertension, or infection). Exclusion criteria were: (1) concomitant intracardiac anomalies (e.g., persistent left superior vena cava or large ventricular septal defects; small ventricular septal defects not visible on grayscale ultrasound were not excluded); (2) fetal growth restriction; (3) chromosomal abnormalities; (4) inadequate image quality; and (5) loss to postnatal follow-up. Inadequate image quality was defined as failure to obtain standard cardiac planes or failure to clearly delineate the foramen ovale flap and left atrial borders for reliable measurement. Postnatal echocardiography and/or pathological examination served as the reference standard. Based on postnatal findings, fetuses with prenatal suspicion of CoA were classified as the false-positive prenatal suspicion group (suspected prenatally but excluded postnatally) or the true CoA group (confirmed both prenatally and postnatally). Fifty gestational age-matched fetuses with normal prenatal findings and normal postnatal follow-up were included as controls. The study was conducted in accordance with the Declaration of Helsinki and was approved by the Ethics Committee of the Second Affiliated Hospital of Fujian Medical University (Approval No. 2023657). Informed consent was waived due to the retrospective design.

### 2.2. Equipment and Ultrasonographic Methods

All prenatal ultrasound examinations were performed according to the Guidelines for Prenatal Ultrasound Examination issued by the Society of Ultrasound in Medicine of the Chinese Medical Doctor Association [12]. Fetal echocardiography followed the recommendations of the International Society of Ultrasound in Obstetrics and Gynecology [13]. Examinations were performed using Voluson 730 Pro, Voluson E8, and Voluson E10 systems (GE Healthcare, Zipf, Austria) with 4–8 MHz transducers. Measurements were obtained as follows. From the apical four-chamber view at end-systole, we measured the maximal diameter of the bulging foramen ovale flap (FOFD), left atrial diameter (LAD), right atrial diameter (RAD), left ventricular diameter (LVD), and right ventricular diameter (RVD). FOFD and LAD were assessed on a standardized apical four-chamber view at end-systole, with adequate magnification and clear visualization of the foramen ovale flap, atrial septum, and left atrial borders. FOFD was defined as the maximal distance from the most prominent edge of the foramen ovale flap to the atrial septal reference line, whereas LAD was defined as the maximal transverse inner-border diameter of the left atrium in the same frame. To improve reproducibility, both measurements were obtained on the same stored image, and repeated measurements were averaged when necessary. Examinations with non-standard sectioning, indistinct anatomical landmarks, or inadequate image quality were considered unsuitable for reliable FOFD/LAD assessment [13,14]. In technically challenging examinations, measurements were attempted only when a standardized apical four-chamber view at end-systole could still be obtained and the foramen ovale flap, atrial septum, and left atrial borders were clearly identifiable on the same stored frame; otherwise, FOFD/LAD was not recorded. From the left ventricular outflow tract view during systole, the aortic diameter (AoD) was measured just above the aortic valve. From the right ventricular outflow tract view during systole, the pulmonary artery diameter (PAD) was measured above the pulmonary valve. From the long-axis view of the aortic arch at end-systole, the maximal diameter of the aortic isthmus was measured, and the aortic isthmus Z-score was calculated using gestational age-specific reference formulas [15,16]. Z-scores were calculated as (expected mean − measured diameter)/SD; therefore, higher Z-scores indicate smaller aortic isthmus diameters.

Examinations were performed by sonographers with at least 3 years of dedicated fetal echocardiography experience, and key measurements were reviewed offline by two observers with ≥5 years of experience. Measurements were obtained using standardized cardiac views and were reviewed offline on stored images. Operators were not aware of the final postnatal diagnosis at the time of image acquisition.

### 2.3. Statistical Analysis

Statistical analyses were performed using R software (version 4.4.1) and IBM SPSS Statistics (version 23.0; IBM Corp., Armonk, NY, USA). Descriptive statistics, group comparisons, correlation analyses and ICC calculations were performed in R. ROC curve analysis was conducted in SPSS. Continuous variables are presented as median (interquartile range). Group comparisons (false-positive prenatal suspicion, true CoA, and control) were performed using the Kruskal–Wallis test. Post hoc pairwise comparisons used the Mann–Whitney U test with Bonferroni correction. An overall two-sided *p* value < 0.05 was considered statistically significant; for post hoc pairwise comparisons, a Bonferroni-adjusted threshold of *p* < 0.017 was applied.

Associations between FOFD/LAD and cardiac structural ratios (RAD/LAD, RVD/LVD, and PAD/AoD) were evaluated using Spearman’s rank correlation. Correlation analysis was performed in the false-positive and control groups to evaluate the association of FOFD/LAD with cardiac structural ratios in fetuses without postnatally confirmed CoA. To visualize potential nonlinear patterns, quadratic curves were plotted for descriptive purposes.

Diagnostic accuracy: Receiver operating characteristic (ROC) curve analysis was performed to evaluate the ability of FOFD/LAD and conventional structural ratios to distinguish false-positive prenatal suspicion from true coarctation of the aorta within a selected cohort of fetuses with prenatal suspicion of CoA. For statistical purposes, false-positive prenatal suspicion was defined as the positive state because the primary aim was to assess recognition of RFOF-related CoA mimicry among fetuses already considered suspicious for CoA. However, the findings should be clinically interpreted as informing the distinction between true CoA and non-CoA/RFOF-related mimicry in this selected diagnostic population. Areas under the curve (AUCs) with 95% confidence intervals were calculated using a nonparametric approach. Optimal cut-off values were determined using the Youden index (sensitivity + specificity − 1), and sensitivity, specificity, PPV, and NPV were reported at the optimal thresholds.

Exploratory multivariable analysis: Because of the limited sample size and the instability of ordinary logistic regression in this dataset, an exploratory Firth penalized logistic regression analysis was additionally performed in the subgroup of fetuses with prenatal suspicion of CoA as a sensitivity analysis adjusting for the aortic isthmus Z-score. This analysis was intended to explore whether FOFD/LAD retained a possible association with group classification; it was not designed to establish definitive independent incremental diagnostic value beyond all conventional markers.

Reproducibility assessment: Intraobserver and interobserver variability were assessed in a randomly selected subset of 20 fetuses. For intraobserver variability, Observer 1 repeated the measurements after a ≥2 week interval and was blinded to the initial results. For interobserver variability, Observer 2 independently measured the same images and was blinded to Observer 1. Reliability was quantified using intraclass correlation coefficients (ICCs) with 95% confidence intervals. Intraobserver reliability was assessed using a two-way mixed-effects model with absolute agreement for single measurements [ICC(3, 1)], whereas interobserver reliability was evaluated using a two-way random-effects model with absolute agreement for single measurements [ICC(2, 1)] (Table S1). In addition, Bland–Altman analysis was performed for FOFD/LAD. For each fetus, the difference between two measurements was plotted against their mean, and the mean difference (bias) and 95% limits of agreement (LOA; bias ± 1.96 SD of the differences) were calculated for both intraobserver and interobserver comparisons (Figure S1).

Because examinations were performed using multiple ultrasound platforms over the study period, some equipment-related measurement variability cannot be excluded.

### 2.4. Use of Generative AI

During manuscript preparation, ChatGPT (OpenAI; model: GPT-5.4) was used to assist with English language editing and improving clarity of the text. The authors reviewed and edited the content and take full responsibility for the integrity of the work and the final manuscript.

## 3. Results

### 3.1. Demographic Characteristics

Among 68 fetuses with prenatal suspicion of CoA, 12 were excluded because of concomitant intracardiac anomalies and/or chromosomal abnormalities, 2 because of fetal growth restriction, and 10 because of loss to follow-up. Overall, 44 fetuses met the inclusion criteria and were enrolled. Postnatally, 29 fetuses did not have CoA (false-positive prenatal suspicion group) and 15 were confirmed to have CoA (true CoA group). The median gestational age at examination was 34 + 3 weeks in the false-positive prenatal suspicion group, 32 + 1 weeks in the true CoA group, and 33 + 4 weeks in controls, with no significant difference among groups (*p* = 0.429).

### 3.2. Correlation Analysis Between FOFD/LAD and Cardiac Structural Parameters

**Figure 1:**
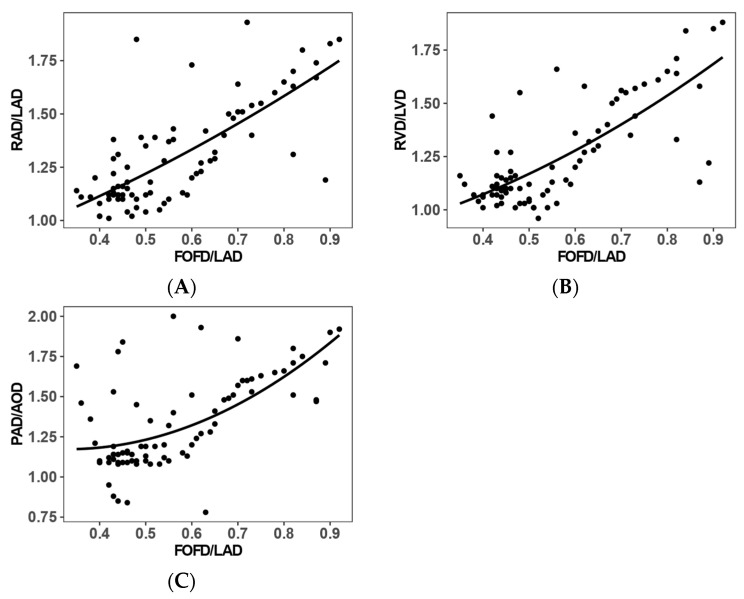
Scatterplots showing the relationships between FOFD/LAD and cardiac structural ratios: (**A**) RAD/LAD, (**B**) RVD/LVD, and (**C**) PAD/AoD. Each dot represents one fetus. The solid lines show fitted quadratic curves for descriptive visualization only and do not represent formal nonlinear modeling.

In the false-positive prenatal suspicion group (*n* = 29) and control (*n* = 50) groups, higher FOFD/LAD values were associated with higher RAD/LAD, RVD/LVD, and PAD/AoD ratios (Figure 1). Spearman correlation coefficients were r = 0.716, 0.666, and 0.591, respectively (all *p* < 0.001). Figure 1 illustrates these relationships.

### 3.3. Comparison of Fetal Cardiovascular Measurements Among the False-Positive Prenatal Suspicion, True CoA, and Control Groups

FOFD/LAD was significantly higher in the false-positive prenatal suspicion group than in the true CoA and control groups (both *p* < 0.001), whereas no significant difference was observed between the true CoA and control groups (*p* = 0.059). Compared with controls, both suspected CoA groups had higher RAD/LAD, RVD/LVD, PAD/AoD ratios, and aortic isthmus Z-scores (all *p* < 0.001). However, RAD/LAD (*p* = 0.843), RVD/LVD (*p* = 0.638), PAD/AoD (*p* = 0.710), and Z-score (*p* = 0.038) did not differ between false-positive prenatal suspicion and true CoA cases (*p* = 0.038 for Z-score did not remain significant after Bonferroni correction; significance threshold: *p* < 0.017) (Table 1).

Receiver operating characteristic (ROC) curve analysis was performed to evaluate the ability of FOFD/LAD and other conventional structural ratios to distinguish false-positive prenatal suspicion of CoA from true CoA within the selected study cohort. FOFD/LAD showed the highest discriminatory performance among the evaluated parameters. However, given the limited number of confirmed CoA cases (*n* = 15), the AUC estimates and proposed optimal cut-off values should be interpreted cautiously as exploratory findings requiring external validation in larger independent cohorts (Table 2).

AUC, area under the curve; CI, confidence interval; PPV, positive predictive value; NPV, negative predictive value. Optimal cut-off values were determined using the Youden index. Test-positive was defined as a value ≥ the cut-off. In an exploratory Firth penalized logistic regression sensitivity analysis, restricted to fetuses with prenatal suspicion of CoA and adjusted only for aortic isthmus Z-score, FOFD/LAD remained associated with group classification. Higher FOFD/LAD values were associated with lower odds of true CoA, whereas higher aortic isthmus Z-scores were associated with higher odds of true CoA. Given the limited sample size and restricted adjustment, these findings should be interpreted as supportive exploratory results rather than definitive evidence of independent, incremental diagnostic value. The corresponding effect estimates were OR 0.116 (95% CI 0.0088–0.3819) per 0.1-unit increase in FOFD/LAD and OR 1.445 (95% CI 1.081–2.369) per 0.1-unit increase in aortic isthmus Z-score.

### 3.4. Reproducibility Analysis of FOFD/LAD

Bland–Altman analysis for FOFD/LAD showed minimal systematic bias in both intraobserver and interobserver assessments. For intraobserver agreement, the mean bias was −0.0068, with 95% limits of agreement ranging from −0.0859 to 0.0723. For interobserver agreement, the mean bias was −0.0060, with 95% limits of agreement ranging from −0.1405 to 0.1285. The wider limits of agreement in the interobserver analysis indicated greater variability between observers than within the same observer. These findings were consistent with the ICC results and support a cautious interpretation of FOFD/LAD in routine practice.

**Figure 2:**
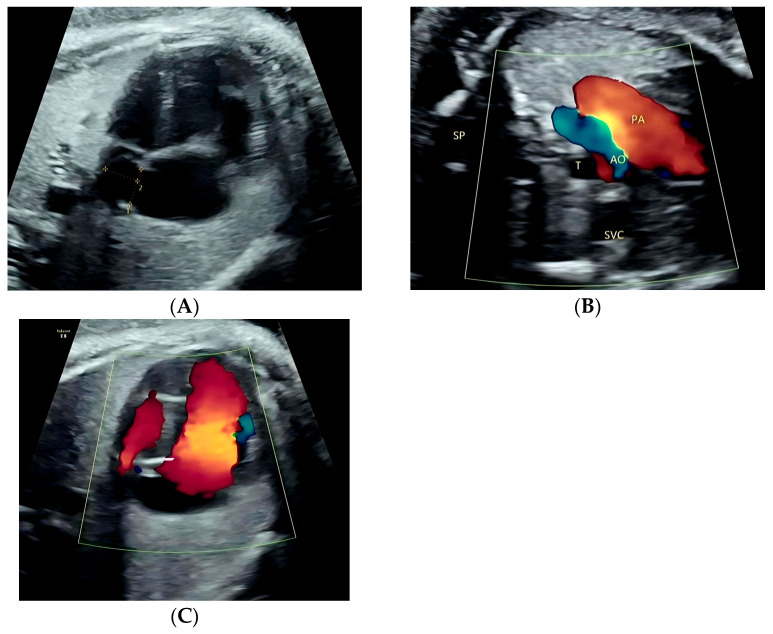
Prenatal ultrasound images from a false-positive case with a redundant foramen ovale flap. (**A**) At 35 + 4 weeks of gestation, the apical four-chamber view shows a redundant foramen ovale flap with mild atrial and ventricular asymmetry. (**B**) Great-vessel view showing disproportion between the pulmonary artery and the aorta. (**C**) Color Doppler apical four-chamber view more clearly demonstrating asymmetric color flow distribution between the right and left heart. **Abbreviations:** PA, pulmonary artery; AO, aorta; SVC, superior vena cava; SP, spine; T, trachea.

**Figure 3:**
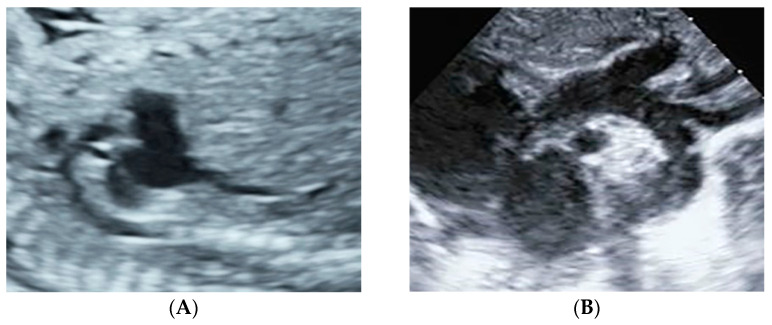
Prenatal and postnatal ultrasound images of the aortic arch. (**A**) At 32 + 6 weeks of gestation, the prenatal scan of the aortic arch showed a stiff and tortuous arch with a narrowed internal diameter, comparable to or slightly smaller than that of the adjacent carotid branches; the measurement yielded an aortic isthmus Z-score of 2.77 based on gestational age reference formulas (higher values indicate smaller isthmus diameters in our Z-score convention); (**B**) Postnatal imaging confirmed normal development of the aortic arch.

Figure 2 illustrates a false-positive case with a redundant foramen ovale flap. The four-chamber view showed only mild right-left asymmetry (Figure 2A), whereas the great-vessel view demonstrated clearer disproportion between the pulmonary artery and the aorta (Figure 2B). In addition, the color Doppler four-chamber view more clearly demonstrated asymmetric color flow distribution between the right and left heart (Figure 2C). Figure 3 shows a fetus with prenatal suspicion of a tortuous and narrowed aortic arch; postnatal echocardiography confirmed normal aortic arch development.

## 4. Discussion

RFOF is uncommon and may be overlooked during prenatal ultrasound, with reported echocardiographic detection rates of approximately 0.6–1.7% [7,17]. In our cohort, higher FOFD/LAD values were associated with more pronounced right-to-left cardiac disproportion, greater pulmonary artery-to-aorta imbalance, and smaller aortic isthmus dimensions (reflected by higher Z-scores in our calculations). Related findings have been reported previously [18,19,20,21,22,23,24,25]. These sonographic patterns resemble commonly reported prenatal markers used to suspect CoA, which is consistent with prior reports [9,18,26,27,28]. Fetal cardiovascular development is influenced by blood volume and shear forces generated by flow [1,2]. From a hemodynamic perspective, an excessively mobile foramen ovale flap may intermittently impede interatrial streaming, partially interfere with mitral inflow, or alter fetal hemodynamics, thereby reducing effective left-heart filling [19,20,21]. Collectively, these mechanisms may help explain why RFOF can mimic CoA-related echocardiographic appearances.

Prenatal ultrasound diagnosis of CoA is associated with a substantial false-positive rate [1,29,30,31]. Wang et al. [22] further highlighted the value of multiparameter assessment in suspected CoA by developing a prenatal ultrasound nomogram for risk stratification; a combined model incorporating aortic isthmus z-score, ascending aorta z-score, pulmonary artery/ascending aorta ratio, persistent left superior vena cava, and aortic arch dysplasia showed excellent diagnostic performance. Stos et al. [23] evaluated 202 fetuses at high risk for CoA and found that a PAD/AoD ratio > 1.6 had limited accuracy (19%). Jung et al. [24] reported that among 44 fetuses with isolated right-dominant hearts and RAD/LAD, RVD/LVD, and PAD/AoD ratios > 1.5, 66% were normal postnatally and 34% had congenital heart disease; CoA and interrupted aortic arch accounted for 27.2%. RFOF may contribute to this diagnostic uncertainty by producing imaging patterns that overlap with suspected CoA [7,9]. The postnatal trajectories of true CoA and RFOF differ. After birth, closure of the foramen ovale and ductus arteriosus and a reduction in pulmonary vascular resistance alter loading conditions. In RFOF, these changes may increase systemic output and facilitate ongoing growth of the aortic arch, whereas in true CoA the arch remains narrowed and may compromise neonatal hemodynamics. Accordingly, careful assessment for RFOF is warranted when CoA is suspected prenatally. These findings should be interpreted in the context of a highly selected diagnostic population, namely fetuses already referred or categorized as having prenatal suspicion of CoA, and should not be directly extrapolated to general fetal anomaly screening populations or to all fetuses with mild left-right asymmetry. Compared with prior studies that mainly described the overlap between RFOF and suspected CoA or emphasized conventional indices of right-left disproportion, the present study specifically evaluated FOFD/LAD as a simple morphology-based adjunctive parameter and examined its relationship with conventional structural markers within a postnatally classified cohort.

The FOFD/LAD ratio should not be interpreted as an independent screening marker or as a standalone diagnostic threshold for CoA. Rather, its likely role is as an adjunctive parameter within the overall fetal echocardiographic assessment in fetuses already considered suspicious for CoA. In clinical practice, this may be particularly relevant in borderline cases with mild ventricular asymmetry, equivocal great-vessel disproportion, or uncertain aortic arch findings, where the differential diagnosis between true CoA and RFOF-related CoA mimicry is being considered.

An increased FOFD/LAD should not be taken in isolation as evidence against true CoA. It may suggest that a redundant foramen ovale flap is contributing to a CoA-like phenotype, especially when the foramen ovale flap appears prominent and mobile and when conventional markers such as right-left chamber disproportion are present but the aortic arch findings are not definitive. In such circumstances, FOFD/LAD may provide additional interpretive value, but it should be integrated with comprehensive assessment of cardiac structure, great-vessel relationships, Doppler findings, and follow-up evaluation.

In this study, false-positive prenatal suspicion and true CoA cases showed similar RAD/LAD, RVD/LVD, PAD/AoD ratios, and aortic isthmus Z-scores, suggesting that these commonly used indices alone may be insufficient to distinguish true CoA from RFOF-related CoA mimicry [26,27,29,30,31]. Although hemodynamic assessment may be helpful, prior work indicates that Doppler features may also overlap between the two entities [18,25]. Notably, FOFD/LAD was higher in the false-positive prenatal suspicion group than in the true CoA group, indicating that FOFD/LAD may be useful as an adjunctive marker for differentiation. However, its reproducibility warrants careful consideration. In the present study, the interobserver ICC for FOFD/LAD was 0.60, indicating only moderate reproducibility. Bland–Altman analysis further showed mean biases close to zero for both intraobserver and interobserver comparisons, suggesting no substantial systematic measurement bias, whereas the limits of agreement were wider for interobserver assessment (−0.1405 to 0.1285) than for intraobserver assessment (−0.0859 to 0.0723). These findings indicate that, although different observers did not show a clear directional bias, between-operator variability at the individual level remained non-negligible. As FOFD/LAD is the principal discriminative parameter in this study, routine applicability may be limited across operators and imaging conditions, and the ratio should be interpreted cautiously in daily practice. This supports the use of FOFD/LAD as an adjunctive rather than a standalone marker and underscores the importance of standardized image acquisition, clearly defined anatomical landmarks, and operator training.

In addition, the applicability of FOFD/LAD depends on adequate image quality. In technically difficult examinations, such as those affected by maternal obesity, unfavorable fetal position, or poor acoustic windows, FOFD/LAD should not be forcibly measured if the foramen ovale flap or left atrial borders cannot be clearly delineated. In such situations, interpretation should rely on the integrated echocardiographic assessment and repeat targeted fetal echocardiographic evaluation when feasible. Therefore, FOFD/LAD should be regarded as a useful adjunct under adequate imaging conditions, but not as a substitute for comprehensive fetal echocardiographic evaluation in technically difficult cases. Marked flap mobility, particularly transient prolapse toward the foramen ovale or mitral orifice, may provide additional diagnostic clues. At present, no standardized sonographic criteria or quantitative thresholds exist for RFOF, and diagnosis relies on an integrated assessment of flap morphology, mobility, and hemodynamic impact. Given the limited number of confirmed CoA cases (*n* = 15), the proposed FOFD/LAD cut-off values and related diagnostic performance metrics should be interpreted as exploratory and require external validation in larger cohorts [26,27,28,29,30,31]; moreover, PPV and NPV are prevalence-dependent and may not directly reflect performance in real-world screening populations.

No standardized management guidelines currently exist for RFOF. In most cases, fetal hemodynamics normalize after birth with closure of the foramen ovale and ductus arteriosus, allowing continued aortic arch growth and generally favorable outcomes. However, because RFOF can be confused with suspected CoA on prenatal imaging, fetuses with prominent RFOF may benefit from closer prenatal surveillance and early postnatal echocardiography. M-mode echocardiography can help assess flap mobility and atrial motion; combining two-dimensional imaging with M-mode may further clarify the hemodynamic significance of RFOF.

This study has several limitations. First, its retrospective single-center design and relatively small sample size, particularly the limited number of confirmed CoA cases, restricts the robustness and generalizability of the findings. Accordingly, the proposed FOFD/LAD cut-off and related diagnostic performance estimates should be regarded as exploratory and hypothesis-generating rather than clinically validated. Second, this was a study of a selected referral cohort of fetuses with prenatal suspicion of CoA rather than of an unselected screening population; therefore, the findings may not be generalizable to routine fetal anomaly screening or to all fetuses with mild cardiac asymmetry. Third, although reproducibility was formally assessed, the interobserver reproducibility of FOFD/LAD was only moderate, which may limit its routine applicability across operators and imaging conditions. Fourth, multivariable assessment was restricted to an exploratory Firth-penalized logistic regression model with a small number of variables because of the limited number of confirmed CoA cases. Simultaneous adjustment for multiple conventional markers of CoA suspicion was not statistically robust in this dataset. Therefore, the apparent incremental value of FOFD/LAD beyond conventional markers should be interpreted cautiously and requires validation in larger independent cohorts. Accordingly, these exploratory findings should not be interpreted as definitive evidence that FOFD/LAD provides independent diagnostic value beyond all conventional echocardiographic markers. Fifth, high-quality Doppler data of aortic arch flow were not available for all cases, precluding direct comparison of hemodynamic differences between RFOF and true CoA. Finally, the use of multiple ultrasound systems over the study period may have introduced measurement variability despite standardized acquisition and reproducibility assessment.

In conclusion, in this selected referral cohort of fetuses with prenatal suspicion of CoA, RFOF may mimic echocardiographic findings associated with suspected CoA and may therefore contribute to false-positive prenatal suspicion. FOFD/LAD, together with assessment of flap morphology and mobility, may provide a useful adjunct for differentiating RFOF-related CoA mimicry from true CoA. These findings remain exploratory and require external validation before clinical application [26,29,31,32].

## Figures and Tables

**Table 1:** Comparison of fetal cardiac structural parameters among three groups.

Group	FOFD/LAD	RAD/LAD	RVD/LVD	PAD/AoD	Z-Score
FP (*n* = 29)	0.72 (0.65, 0.82)	1.54 (1.40, 1.70)	1.55 (1.36, 1.61)	1.60 (1.51, 1.71)	3.00 (2.50, 3.20)
TC (*n* = 15)	0.43 (0.40, 0.47)	1.52 (1.40, 1.71)	1.50 (1.42, 1.54)	1.60 (1.48, 1.71)	3.20 (3.05, 3.40)
N (*n* = 50)	0.46 (0.43, 0.53)	1.12 (1.10, 1.22)	1.10 (1.04, 1.15)	1.13 (1.09, 1.20)	0.22 (−0.18, 0.38)

Overall *p* values are reported in the text. *p* values reflect overall group comparisons (Kruskal–Wallis test). Post hoc pairwise comparisons were performed using the Mann–Whitney U test with Bonferroni correction (adjusted significance threshold *p* < 0.017). FP, false-positive prenatal suspicion; TC, true CoA; N, control.

**Table 2:** ROC analysis for identifying false-positive prenatal suspicion of CoA (positive state).

Variable	AUC	95%CI	*p* Value	Optimal Cut-Off (≥)	Sensitivity	Specificity	PPV	NPV
FOFD/LAD	0.933	0.855–1.000	<0.001	0.610	0.828 (24/29)	1.000 (15/15)	1.000 (24/24)	0.750 (15/20)
PAD/AoD	0.536	0.346–0.725	0.701	1.405	0.966 (28/29)	0.200 (3/15)	0.700 (28/40)	0.750 (3/4)
RVD/LVD	0.545	0.372–0.718	0.629	1.540	0.552 (16/29)	0.733 (11/15)	0.800 (16/20)	0.458 (11/24)
RAD/LAD	0.480	0.297–0.663	0.833	1.475	0.655 (19/29)	0.400 (6/15)	0.679 (19/28)	0.375 (6/16)

## Data Availability

The datasets used and/or analyzed during the current study are available from the corresponding author upon reasonable request.

## Supplementary Materials

The following supporting information can be downloaded at: https://www.mdpi.com/article/10.3390/jcm15083166/s1, Figure S1: Bland–Altman plots for FOFD/LAD reproducibility. (A) Intraobserver agreement between the first and second measurements obtained by Observer 1. (B) Interobserver agreement between measurements obtained by Observer 1 and Observer 2. The solid line represents the mean difference (bias), and the dashed lines represent the 95% limits of agreement; Table S1: Intra- and interobserver reproducibility (ICC) for fetal echocardiographic measurements.
