# Supplementary material for: Ultrasound Characteristics of an Isolated Redundant Foramen Ovale Flap in Fetuses and Its Differential Diagnosis from Aortic Coarctation
Source: J Clin Med. 2026 Apr 21;15(8):3166. doi: 10.3390/jcm15083166 (PMC13117341; doi:10.3390/jcm15083166)
Supplement: Supplementary file 1 [file jcm-15-03166-s001.zip › jcm-4149135-Supplementary.pdf]

Supplementary Materials:

Table S1. Intra- and interobserver reproducibility (ICC) for fetal echocardiographic measurements

Variable	Intraobserver ICC (95% CI)	Interobserver ICC (95% CI)
FOFD	0.94(0.86–0.98)	0.89(0.74–0.95)
LAD	0.98(0.95–0.99)	0.87(0.70–0.95)
RAD	0.98(0.96–0.99)	0.85(0.65–0.94)
LVD	0.96(0.91–0.98)	0.80(0.57–0.92)
RVD	0.98(0.96–0.99)	0.91(0.78–0.96)
AoD	0.91(0.79–0.96)	0.81(0.58–0.92)
PAD	0.93(0.82–0.97)	0.82(0.61–0.93)
AID	0.94(0.85–0.97)	0.88(0.72–0.95)
FOFD/LAD	0.87(0.71 - 0.95)	0.60(0.24 - 0.82)

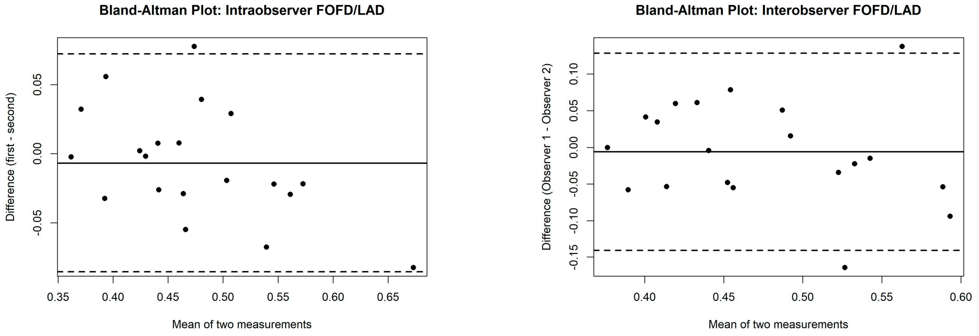

(A) (B)

Figure S1. Bland–Altman plots for FOFD/LAD reproducibility.
(A) Intraobserver agreement between the first and second measurements obtained by Observer 1.
(B) Interobserver agreement between measurements obtained by Observer 1 and Observer 2.
The solid line represents the mean difference (bias), and the dashed lines represent the 95% limits of agreement.
